# MiRNA-203 suppresses tumor cell proliferation, migration and invasion by targeting Slug in gastric cancer

**DOI:** 10.1007/s13238-016-0259-4

**Published:** 2016-03-22

**Authors:** Liuqing Yang, Hongwei Liang, Yanbo Wang, Shanting Gao, Kai Yin, Zhijian Liu, Xi Zheng, Ying Lv, Lei Wang, Chen-Yu Zhang, Xi Chen, Guifang Xu, Weijie Zhang, Xiaoping Zou

**Affiliations:** Department of Gastroenterology, Drum Tower Hospital, Medical School of Nanjing University, Nanjing, 210008 China; State Key Laboratory of Pharmaceutical Biotechnology, Collaborative Innovation Center of Chemistry for Life Sciences, Jiangsu Engineering Research Center for MicroRNA Biology and Biotechnology, NJU Advanced Institute for Life Sciences (NAILS), School of Life Sciences, Nanjing University, Nanjing, 210023 China; Department of General Surgery, Drum Tower Hospital, Medical School of Nanjing University, Nanjing, 210008 China; Department of General Surgery, Drum Tower Clinical College of Nanjing Medical University, Nanjing, 210018 China

**Dear Editor,**

Snail, a family of zinc finger transcription factors, plays an important role in morphogenesis and embryogenesis. Snail zinc finger family 2 (SNAI2 or Slug) has been demonstrated to regulate carcinogenesis of several human cancers including breast, prostate, head, neck, pancreas and endometrial carcinomas (Zhang et al., 2011; Markiewicz et al., 2012; Behnsawy et al., 2013; Smith et al., 2013; Tanaka et al., 2013), and it contributes to various tumorigenesis processes ranging from tumor cell invasion and metastasis to cell survival and proliferation (Phillips and Kuperwasser, 2014; Shi et al., 2015). However, its participation in the carcinogenesis of GC has only a few studies. Moreover, the molecular mechanisms of upstream and downstream regulation of Slug are largely unknown. Recently, Shi et al. confirmed miR-203 suppression in GC promotes Slug-mediated cancer metastasis (Shi et al., 2015). In this study, we explored the relationship of Slug and miRNAs using bioinformatics analysis, and demonstrated the roles of candidate miRNA, miR-203, in the carcinogenesis of gastric cancer cells.

We first examined Slug expression in gastric cancer tissues by Western blotting. As shown in Fig. 1A, the Slug protein levels were significantly increased in the gastric cancer tissues compared to the corresponding adjacent normal tissues, suggesting that Slug may serve as an oncogene in the gastric cancer. In contrast, the Slug mRNA levels did not significantly differ between cancerous and noncancerous tissues (Fig. 1B). The disparity between Slug protein and mRNA expression in gastric cancer vs. adjacent normal tissues suggests that Slug may be regulated through a post-transcriptional mechanism.

**Figure 1:**
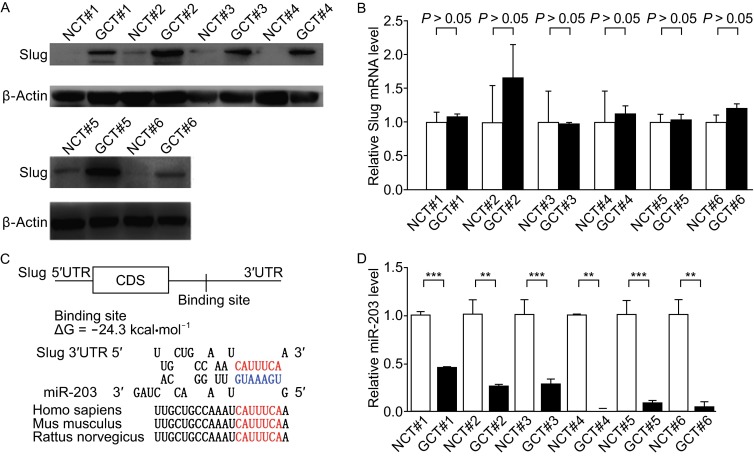
**Upregulation of Slug protein but not mRNA expression and downregulation of miR-203 in human gastric cancer tissues**. (A) Western blotting analysis of the expression levels of Slug protein in 6 pairs of GCT and NCT samples. (B) Quantitative RT-PCR analysis of the relative expression levels of Slug mRNA in 6 pairs of gastric cancer tissue (GCT) and noncancerous tissue (NCT) samples. (C) Schematic depicting the hypothetical duplexes formed through interactions between the binding sites in the Slug 3′-UTR (top) and miR-203 (bottom). The predicted free energy of each hybrid is indicated. The seed recognition sites are denoted, and all nucleotides in these regions are highly conserved across species. (D) Quantitative RT-PCR analysis of the miR-203 expression levels in 6 pairs of GC and GN samples. *P < 0.05; **P < 0.01; ***P < 0.001

MiRNAs mediate post-transcriptional regulation by repressing mRNA transcription. We used three computational algorithms, including TargetScan (Lewis et al., 2003), miRanda (John et al., 2004) and PicTar (Krek et al., 2005), to investigate miRNAs that can potentially target Slug, and miR-203 was identified as the candidate regulator of Slug. The predicted interaction between miR-203 and the target sites in the Slug 3′-UTR are illustrated in Fig. 1C. One predicted hybridization was identified between miR-203 and the 3′-UTR of Slug. The minimum free energy values of the two hybridizations were −24.3 kcal·mol^−1^, and the value was well within the range of genuine miRNA-target pairs. Moreover, the miR-203 binding sequences in the Slug 3′-UTR are highly conserved across species.

MiRNAs and their targets are usually thought to express in an opposite pattern in tissues. We next investigated whether miR-203 was inversely correlated with Slug in gastric cancer tissues. We thus examined the miR-203 levels in the same six pairs of gastric cancer tissues and noncancerous tissues, and observed that miR-203 levels were indeed decreased in gastric cancer tissues as compared to those in adjacent normal tissues (Fig. 1D). These results implied a miR-203-mediated post-transcriptional regulatory mechanism in Slug repression.

The correlation between miR-203 and Slug was further examined after evaluating Slug expression in the human gastric carcinoma cell line MKN-45 after the overexpression or knockdown of miR-203. Overexpression was achieved after transfecting the cells with miR-203 mimic, a synthetic RNA oligonucleotide that mimics the miR-203 precursor, and knockdown was achieved after transfecting cells with miR-203 inhibitor, a chemically modified antisense oligonucleotide designed to specifically target mature miR-203. The transfection efficiency was presented in Fig. 2A. As anticipated, overexpressing miR-203 significantly increased miR-203 levels and suppressed the Slug protein levels in MKN-45 cells, whereas miR-203 knockdown had the opposite effect on MKN-45 expression in these cells (Fig. 2B). We also examined the expression of the Slug mRNA levels, and found overexpression or knockdown of miR-203 did not affect Slug mRNA levels (Fig. 2C). Furthermore, we found that, overexpression of miR-203 also significantly decreased Slug protein levels in AGS cells (Fig. S1).

**Figure 2:**
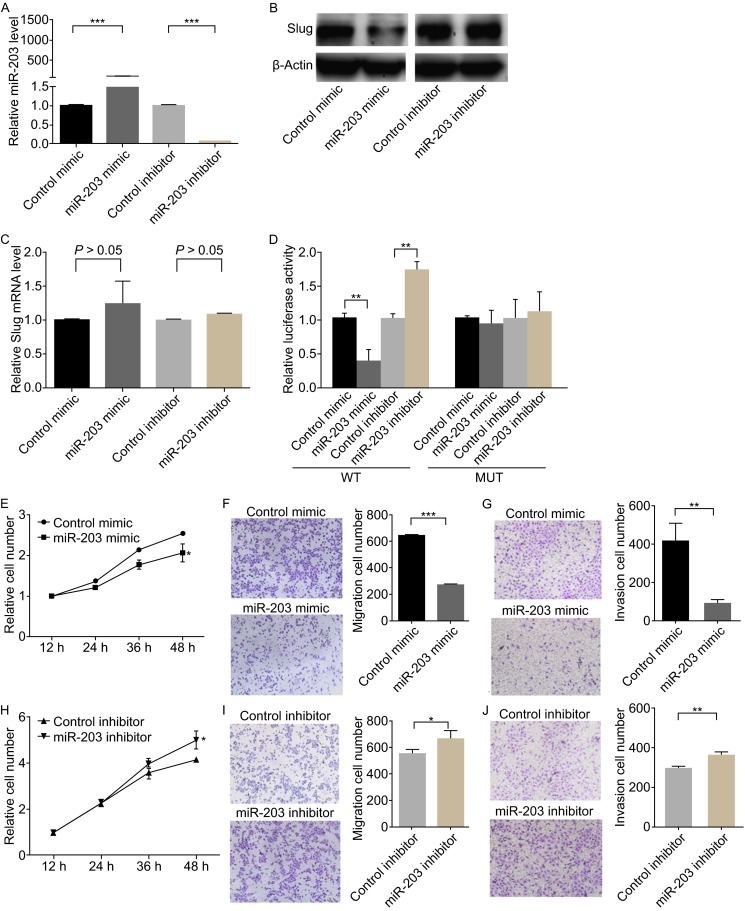
**miR-203 might inhibit cell proliferation, migration and invasion through silencing Slug**. (A) Quantitative RT-PCR analysis of miR-203 levels in MKN-45 cells transfected with miR-203 mimic or inhibitor. (B) Western blotting analysis of Slug protein levels in MKN-45 cells transfected with miR-203 mimic or inhibitor. (C) Quantitative RT-PCR analysis of Slug mRNA levels in MKN-45 cells transfected with miR-203 mimic or inhibitor. (D) Direct recognition of the Slug 3′-UTR by miR-203. Firefly luciferase reporters containing either wild-type (WT) or mutant (MUT) miR-203 binding sites in the Slug 3′-UTR were co-transfected into MKN-45 cells with the scrambled negative control RNA, miR-203 mimic or inhibitor. 24 h post-transfection, the cells were assayed using a luciferase assay kit. The results are calculated as the ratio of firefly luciferase activity in the miR-203 transfected cells normalized to the control cells. (E) Cell proliferation assay was performed 12, 24, 36 and 48 h after the transfection of MKN-45 cells with control mimic or miR-203 mimic. (F) Left panel: Representative image of Transwell migration assay analysis of MKN-45 cells that were transfected with control mimic or miR-203 mimic. Right panel Quantitative analysis of the migration rates. (G) Left panel: Representative image of Transwell invasion assay analysis of MKN-45 cells that were transfected with control mimic or miR-203 mimic. Right panel: Quantitative analysis of the invasion rates. (H) Cell proliferation assay was performed 12, 24, 36 and 48 h after the transfection of MKN-45 cells with control inhibitor or miR-203 inhibitor. (I) Left panel: Representative image of Transwell migration assay analysis of MKN-45 cells that were transfected with control inhibitor or miR-203 inhibitor. Right panel: Quantitative analysis of the migration rates. (J) Left panel: Representative image of Transwell invasion assay analysis of MKN-45 cells that were transfected with control inhibitor or miR-203 inhibitor. Right panel: Quantitative analysis of the invasion rates.*P < 0.05; **P < 0.01; ***P < 0.001

To determine whether the negative regulatory effects of miR-203 on Slug expression were mediated through binding of miR-203 to the presumed sites in the 3′-UTR of the Slug mRNA, the full-length 3′-UTR of Slug was placed downstream of the firefly luciferase gene in a reporter plasmid. The resulting plasmid was transfected into MKN-45 cells along with miR-203 mimic, miR-203 inhibitor or scrambled negative control RNAs. Luciferase activity was markedly decreased in cells transfected with miR-203 mimic and increased in the cells transfected with miR-203 inhibitor (Fig. 2D). Moreover, we introduced point mutations into the corresponding complementary sites in the 3′-UTR of Slug to eliminate the predicted miR-203 binding sites. This mutated luciferase reporter was unaffected through either the overexpression or knockdown of miR-203 (Fig. 2D). This finding suggested that the binding sites contribute to the interaction between miR-203 and Slug mRNA. In conclusion, the results demonstrate that miR-203 inhibits Slug expression by binding to the 3′-UTR of Slug mRNA transcript and inhibits Slug translation.

Slug has been reported to involve in a diverse number of processes ranging from tumor cell invasion and metastasis to cell survival and proliferation (Phillips and Kuperwasser, 2014). To investigate the cellular phenotypes triggered by the miR-203 mediated downregulation of Slug, MKN-45 cells were transfected with either miR-203 mimic, miR-203 inhibitor or si-Slug and analyzed for changes in cell proliferation, migration and invasion.

Firstly, we investigated the role of Slug in proliferation, migration and invasion of gastric cancer cell lines. To knock down Slug, a siRNA targeting Slug was designed and transfected into MKN-45 cells. The efficient knockdown of Slug in MKN-45 cells is shown in Fig. S2A and S2B. MKN-45 cells transfected with Slug siRNA showed decreased cell proliferation, migration and invasion (Fig. S2C–G). Subsequently, we evaluated the effects of miR-203 on the cell proliferation, migration and invasion of MKN-45 cells by repressing Slug. As expected, MKN-45 cells transfected with miR-203 showed decreased proliferation, migration and invasion (Fig. 2E–G); in contrast, knocking down miR-203 with miR-203 inhibitor enhanced cell proliferation, migration and invasion (Fig. 2H–J).

Taken together, we confirmed that Slug expression is up-regulated in gastric cancer tissues and plays a key function in gastric cancer cells proliferation, migration and invasion. We also identified Slug as a novel target of miR-203 in gastric cancer. These data suggest that Slug and miR-203 expression might be useful to predict invasiveness of gastric cancer, or be used as a prognostic factor in gastric cancer patients. The potential of Slug or miR-203 as novel molecular targets for gastric cancer therapy requires further investigations.

## Electronic supplementary material

Below is the link to the electronic supplementary material.

Supplementary material 1 (PDF 323 kb)
