# Supplementary material for: MiRNA-203 suppresses tumor cell proliferation, migration and invasion by targeting Slug in gastric cancer
Source: Protein Cell. 2016 Mar 22;7(5):383–7. doi: 10.1007/s13238-016-0259-4 (PMC4853317; doi:10.1007/s13238-016-0259-4)

# Supplementary Data

## Supplementary materials and methods

### Cells and human tissues

The human gastric cancer cell lines AGS and MKN-45 were purchased from the Shanghai Institute of Cell Biology, Chinese Academy of Sciences (Shanghai, China). MKN-45 and AGS were cultured in RPMI 1640 medium supplemented with 10% FBS (GIBCO, CA, USA). The cells were incubated at 37 ℃ in a humidified atmosphere of 5% $CO_2$. Gastric cancer tissue and paired normal adjacent tissues were derived from patients undergoing a surgical procedure at the Affiliated Drum Tower Hospital of Nanjing University (Nanjing, China). A signed consent form was obtained from each donor. The tissue fragments were immediately frozen in liquid nitrogen at the time of surgery and stored at -80 ℃. The clinical features of the patients are listed in Supplementary Table 1. The study protocols were approved by Medical Ethics Committee of the Affiliated Drum Tower Hospital of Nanjing University (Nanjing, China); and all experiments were performed in accordance with approved guidelines of the Affiliated Drum Tower Hospital of Nanjing University (Nanjing, China).

### Overexpression or knockdown of miR-203

Synthetic miR-203 mimic, inhibitor and scrambled negative control RNA (control mimic and control inhibitor) were purchased from Genepharma (Shanghai, China). MKN-45 cells or AGS cells were seeded in 6-well plates and than transfected with Lipofectamine 2000 (Invitrogen) on the following day when the cells were approximately 70% confluent. In each well, equal amounts (100 pmol) of miR-203 mimic, inhibitor or the scrambled negative control RNAs were used. The cells were harvested 24 h after transfection for quantitative RT-PCR and 48 h for Western blotting.

### siRNA interference assay

Three siRNA sequences targeting different sites of the human Slug cDNA (Slug siRNA) were designed and synthesized by RIBOBIO. A scrambled siRNA that did not target the human Slug cDNA was synthesized as a negative control. The Slug siRNA were transfected into MKN-45 cells using Lipofectamine 2000 (Invitrogen) according to the manufacturer’s instructions. RNA was extracted 24 hours after transfection while proteins were isolated 48 hours post-transfection. The Slug mRNA and protein expression levels were assessed by quantitative RT-PCR and Western blotting. The siRNA sequence with the best interfering effect (si-Slug #1) was selected and used in this study.

### RNA isolation and quantitative RT-PCR

Total RNA was extracted from the cultured cells and human tissues using TRIzol Reagent (Invitrogen, Carlsbad, CA) according to the manufacturer’s instructions. Assays to quantify miRNAs were performed using Taqman miRNA probes (Applied Biosystems, Foster City, CA) according to the manufacturer’s instructions. Briefly, 1 μg of total RNA was reverse-transcribed to cDNA using AMV reverse transcriptase (TaKaRa, Dalian, China) and a stem-loop RT primer (Applied Biosystems). The reaction conditions were as follows: 16 ℃ for 30 min, 42 ℃ for 30 min, and 85 ℃ for 5 min. Real-time PCR was performed using a TaqMan PCR kit on an Applied Biosystems 7300 Sequence Detection System (Applied Biosystems). The reactions were incubated in a 96-well optical plate at 95 ℃ for 10 min, followed by 40 cycles of 95 ℃ for 15 sec and 60 ℃ for 1 min. All of the reactions were run in triplicate. After the reaction, the cycle threshold ($C_T$) data were determined

using fixed threshold settings, and the mean $C_T$ of the triplicate PCRs was determined. A comparative $C_T$ method was used to compare each condition to the controls. The relative levels of the miRNAs in cells and tissues were normalized to U6. The amount of miRNA relative to the internal control U6 was calculated using the $2^{-\triangle\triangle CT}$ equation, in which $\triangle\triangle C_T$ = ($C_T$ miRNA - $C_T$ U6)target - ($C_T$ miRNA - $C_T$ U6)control.

To quantify Slug mRNA, 1 μg of total RNA was reverse-transcribed to cDNA using oligo dT and Thermoscript (TaKaRa) in the reaction, which was performed with the following conditions: 16 ℃ for 30 min , 42 ℃ for 30 min and 85 ℃ for 5 min. Next, real-time PCR was performed using the RT product, SYBER Green Dye (Invitrogen), and specific primers for Slug and β-actin. The sequences of the primers were as follows:

Slug (R): 5'-GTGTTTGCAAGATCTGCGGC-3';
Slug (F): 5'-GAGCCCTCAGATTTGACCTGT -3';
β-actin (R): 5'-ACTCGTCATACTCCT GCT-3';
And β-actin (F): 5'-ACAGGATGCAGAAGG AGATAC-3'.

The reactions were incubated at 95 ℃ for 5 min, followed by 40 cycles of 95 ℃ for 30 sec, 55 ℃ for 30 sec, and 72 ℃ for 30 sec. After the reactions were completed, the $C_T$ values were determined by setting a fixed threshold. The relative amount of Slug mRNA was normalized to β-actin.

**Protein extraction and Western blotting**

Protein was extracted from the cultured cells and human tissues using RIPA Lysis buffer (Beyotime, Shanghai, China) according to the manufacturer's instructions. Briefly, the cells and tissues were lysed in RIPA Lysis buffer (Beyotime, Shanghai, China) supplemented with a Protease and Phosphatase Inhibitor Cocktail (Thermo Scientific 78440) on ice for 30 min and then centrifuged for 10 min (12,000 x g, 4 ℃). The supernatant was collected, and the protein concentration was calculated with a Pierce BCA protein assay kit (Thermo Scientific, Rockford, IL, USA). The Slug protein levels were analyzed by Western blotting with a monoclonal anti-human Slug antibody (9585S,CST) .The protein levels were normalized by probing the same blots with β-actin antibody (Mab1445,MULTI). The anti-Slug and anti-β-actin antibodies were purchased from Santa Cruz Biotechnology (CA, USA).

**Luciferase reporter assay**

To test the direct binding of miR-203 to the target gene Slug, a luciferase reporter assay was performed according to the manufacturer's instructions. A sequence containing the presumed miR-203 binding site was designed from the human Slug 3'-untranslated region (3'-UTR). The sequence was inserted into the p-MIR-reporter plasmid (Ambion). The insertion was confirmed to be correct by sequencing. To test the binding specificity, the sequences that interacted with the miR-203 seed sequence were mutated, and the mutant Slug 3'-UTR was inserted into an equivalent luciferase reporter. For the luciferase reporter assays, MKN-45 cells were cultured in 24-well plates, and each well was transfected with 0.4 μg of firefly luciferase reporter plasmid, 0.4μg of a β-galactosidase (β-gal) expression plasmid (Ambion), and equal amounts (20 pmol) of pre-miR-203, anti-miR-203, or the scrambled negative control RNAs using Lipofectamine 2000 (Invitrogen). The β-gal plasmid was used as a transfection control. Twenty-four hours post-transfection, the cells were assayed using a luciferase assay kit (Promega, Madison, WI, USA).

**Cell proliferation assay**

MKN-45 cells were seeded at $7\times10^3$ cells per well on 96-well plates and then incubated overnight in RPMI-1640 supplemented with 10% FBS. The cells were collected at 12, 24, 36, or 48h post-transfection. After transfection, 10 μL of Cell Counting Kit-8 solution (CK04-500, Dojindo) was added to the corresponding tested wells and incubated for 2h. The absorbance was measured at a wavelength of 450 nm. All experiments were performed in triplicate.

**Cell migration and invasion assay**

The migration and invasion ability of MKN-45 cells transfected with miR-203 mimic, miR-203 inhibitor or the Slug siRNA was tested in a Transwell Boyden Chamber (8 mm, Costar, USA). The polycarbonate membranes on the bottom of the upper compartment of the Transwell were coated with 1% human fibronectin (R&D systems 1918-FN, USA). The cells were harvested 12h after transfection and suspended in fetal bovine serum (FBS)-free 1640 culture medium. Then, cells were added to the upper chamber ($6\times10^4$ cells/well). At the same time, 0.6 ml of 1640 with 20% FBS was added to the lower compartment, and the Transwell-containing plates were incubated for 24h in a 5% $CO_2$ atmosphere saturated with $H_2O$. After incubation, cells that had entered the lower surface of the filter membrane were fixed with 4% paraformaldehyde for 25 min at room temperature, washed 3 times with distilled water and stained with 0.5% crystal violet at room temperature. Cells remaining on the upper surface of the filter membrane were scraped off gently with a cotton swab. The lower surfaces were captured by a photomicroscope (five fields per chamber) (BX51 Olympus,Japan), and the cells were counted carefully.

**Statistical analysis**

All Western blot images are representative of at least three independent experiments. Quantitative RT-PCR, luciferase reporter assays, and cell apoptosis assays were performed in triplicate, and each experiment was repeated several times. The data are shown as the means ± SE of at least three independent experiments. The differences were considered statistically significant at $p < 0.05$ using Student's t-test.

**Supplementary Table 1. Demographic information of gastric carcinoma patient cohort.**

| | Gender | Age | Pathological Stage |
|---|---|---|---|
| case#1 | Male | 63 | ⅢA（T3,N2,cM0） |
| case#2 | Male | 61 | ⅢA（T3,N2,cM0） |
| case#3 | Male | 56 | ⅡB（T4a,N0,cM0） |
| case#4 | Female | 46 | ⅢB（T4a,N2,cM0） |
| case#5 | Male | 50 | ⅢC（T3,N3,cM0） |
| case#6 | Female | 66 | IIIC（T4,N3, cM0） |

**Supplementary Figure 1. Downregulation of Slug by miR-203 in AGS cells. (A)** Quantitative RT-PCR analysis of miR-203 levels in AGS cells treated with control mimic, or miR-203 mimic. **(B)** Western blotting analysis of Slug protein levels in AGS cells treated with control mimic, or miR-203 mimic. *** $P < 0.001$.

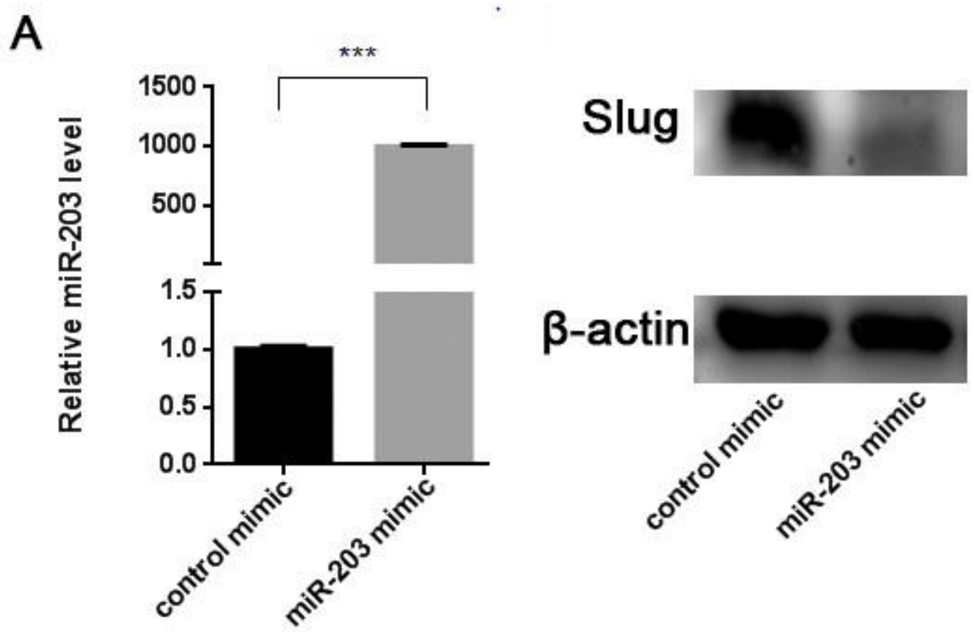

**Supplementary Figure 2: The effect of Slug on the proliferation, migration and invasion of gastric cancer cells. (A)** Quantitative RT-PCR analysis of Slug mRNA levels in MKN-45 cells transfected with control siRNA or Slug siRNA. **(B)** Western blotting analysis of Slug protein levels in MKN-45 cells transfected with control siRNA or Slug siRNA. **(C)** Cell proliferation assay was performed 12h, 24h, 36h and 48h after the transfection of MKN-45 cells with control siRNA or Slug siRNA. **(D)** Representative image of Transwell migration assay analysis of MKN-45 cells that were transfected with control siRNA or Slug siRNA. **(E)** Quantitative analysis of the migration rates. **(F)** Representative image of Transwell invasion assay analysis of MKN-45 cells that were transfected with control siRNA or Slug siRNA. **(G)** Quantitative analysis of the invasion rates. * $P < 0.05$; *** $P < 0.001$.

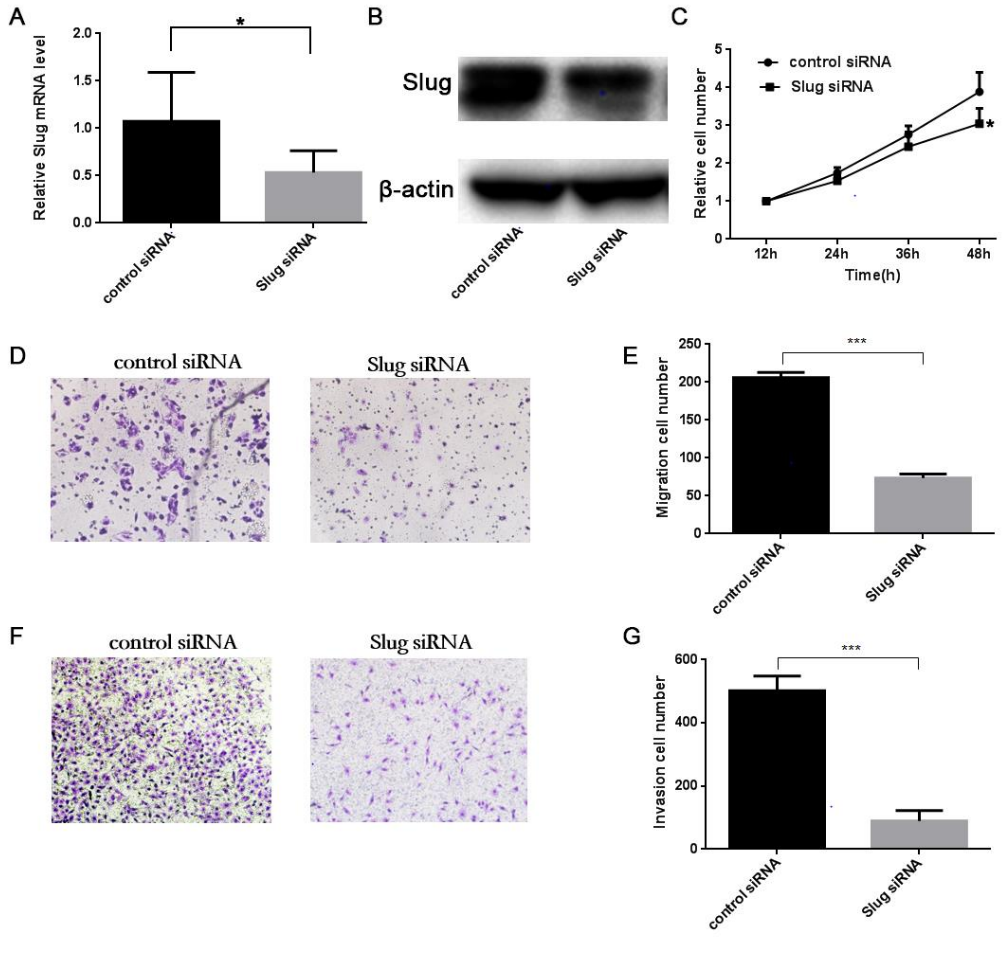

Supplement: Supplementary file 1 — Supplementary material 1 (PDF 323 kb) [file 13238_2016_259_MOESM1_ESM.pdf]
